# Worse outcome of debridement, antibiotics, and implant retention in acute hematogenous infections than in postsurgical infections after total knee arthroplasty: a multicenter study

**DOI:** 10.1186/s43019-022-00165-z

**Published:** 2022-08-17

**Authors:** Moon Jong Chang, Du Hyun Ro, Tae Woo Kim, Yong Seuk Lee, Hyuk-Soo Han, Chong Bum Chang, Seung-Baik Kang, Myung Chul Lee

**Affiliations:** 1 Department of Orthopedic Surgery, Seoul National University College of Medicine, SMG-SNU Boramae Medical Center, Seoul, Republic of Korea; 2 Department of Orthopedic Surgery, Seoul National University College of Medicine, Seoul National University Hospital, 101 Daehak-ro, Jongno-gu, Seoul, 110-744 Republic of Korea; 3 Department of Orthopedic Surgery, Seoul National University College of Medicine, Seoul National University Bundang Hospital, Seongnam, Republic of Korea

**Keywords:** Total knee arthroplasty, Periprosthetic joint infection, Acute postsurgical infection, Acute hematogenous infection

## Abstract

**Background:**

We sought to determine (1) the success rate of debridement, antibiotics, and implant retention (DAIR) for acute periprosthetic joint infection (PJI) of the knee in patients with acute postsurgical infection and in those with acute hematogenous infection via a multicenter study, (2) the factors related to the failure of DAIR for overall acute PJI and acute hematogenous PJI via subgroup analysis, and (3) whether the PJI recurrence patterns differed between the two groups over time after DAIR.

**Methods:**

This retrospective multicenter study included 101 acute knee PJI. Acute postsurgical PJI was defined as PJI diagnosed < 3 months following initial knee arthroplasty surgery. DAIR was performed for 34 cases of acute postsurgical PJIs (postsurgical group) and 67 cases of acute hematogenous PJIs (hematogenous group). The success rates between groups were compared, and factors related to DAIR failure were analyzed.

**Results:**

The overall success rate of DAIR was 77%. The success rate tended to be higher in the postsurgical group than in the hematogenous group (*p* = 0.060). However, there was no significant factor related to DAIR failure in the subgroup analysis of acute hematogenous PJIs. In the postsurgical group, the recurrence of PJI occurred until 3 months, whereas in the hematogenous group, recurrence occurred for up to 2 years.

**Conclusions:**

The failure rate tended to be higher in the acute hematogenous PJI group than in the acute postsurgical PJI group. Since acute hematogenous infections may recur for a longer period than postsurgical infections, careful follow-up is required after DAIR.

## Background

Periprosthetic joint infections (PJIs) are one of the most serious complications after total knee arthroplasty (TKA) [1–4]^)^. In the early stage of a PJI, debridement, antibiotics, and implant retention (DAIR) may be useful surgical options [5, 6]. If a PJI can be treated using DAIR, this can substantially reduce the surgical morbidity of the patient [7]. However, the existing success rate of DAIR ranges widely from 16% to 82% [8, 9]. Furthermore, a previous study revealed that failed prior DAIR could lead to an increased failure rate of subsequent two-stage revision arthroplasty [10]. Therefore, DAIR should be used for reasonable indications.

Despite the improvements in surgical techniques and patient care, the success rate of DAIR has not improved significantly [11]. Although the indications or guidelines for DAIR have not yet been clearly established, patient selection is probably the most important factor for the success of DAIR [12–14]. For patient selection, surgeons need to understand the factors related to DAIR failure. Patient age, immune status, obesity, and comorbid diseases are all likely to be involved but the impact of these factors has been inconsistently reported depending upon the study [12, 14–16]. Recently, it was revealed that the rate of DAIR failure in acute hematogenous PJI was almost two times higher than that in acute postsurgical PJI [17]. In acute postsurgical PJI, it is reasonable that DAIR should be selected as the first treatment method because it is clear when the infection began. However, a different surgical strategy is probably needed for acute hematogenous PJI according to the patients’ conditions. In addition, sometimes it is not clear when the infection started in acute hematogenous PJI. If postsurgical and hematogenous PJIs have different characteristics, the factors related to DAIR failure may be different for acute postsurgical and acute hematogenous PJIs. However, most previous studies included both acute postsurgical and acute hematogenous PJIs in the same category of acute infections for analyzing the factors related to DAIR failure.

Therefore, we sought to determine (1) the success rate of DAIR for acute PJI of the knee in patients with acute postsurgical and acute hematogenous infections using a multicenter study, (2) the factors related to the failure of DAIR for overall acute PJI and acute hematogenous PJI using subgroup analysis, and (3) whether the PJI recurrence patterns differed between acute postsurgical and acute hematogenous PJIs over time after DAIR. We hypothesized that the DAIR failure rate would be higher in acute hematogenous PJI than in acute postsurgical PJI. We also hypothesized that the risk factors associated with DAIR failure and the recurrence patterns would differ between acute postsurgical and acute hematogenous PJIs.

## Methods

This retrospective multicenter study included 101 knees (95 patients) from three referral hospitals between July 2005 and November 2019. The inclusion criteria for this study were as follows: (1) PJI after primary and revision TKA; (2) DAIR with polyethylene insert changes; and (3) acute postsurgical infection and acute hematogenous infection within 4 weeks of the onset of symptoms. 102 cases were found using all criteria 1, 2, and 3 of PJIs were identified via review of medical records. Before 2011, PJI was diagnosed using various criteria, and after 2011, it was diagnosed using Musculoskeletal Infection Society (MSIS) PJI diagnosis criteria [18, 19]. Among these cases, 36 PJIs were diagnosed before 2011. For cases diagnosed before 2011, only patients who could be diagnosed with PJI were included by applying the 2011 MSIS criteria retrospectively. Finally, 101 knees were included in this study after excluding 1 case of PJI that did not meet MSIS criteria. The patients were classified as having acute postsurgical PJI if the PJI was diagnosed < 3 months following the initial knee arthroplasty surgery. An acute hematogenous infection was defined as PJI diagnosed after more than 3 months had passed following the index arthroplasty [17]. Patients with revision arthroplasty were also included in the study, but only if they had aseptic conditions before revision surgery, determined via laboratory tests, joint aspiration, and intraoperative cultures. To include only patients defined as having acute infections, patients with DAIR for an uncertain duration of symptoms were excluded. There were 81 female and 14 male patients with a mean age of 74 years (range 61–86 years). DAIR was performed for 34 cases of acute postsurgical PJI (postsurgical group) and 67 cases of acute hematogenous PJI (hematogenous group) (Table 1). The mean duration of symptoms before DAIR was 6.8 days in the postsurgical group and 8.3 days in the hematogenous group. Average body mass index (BMI) and incidence of diabetes mellitus (DM) were higher in the hematogenous group than in the postsurgical group (58% versus 26%). The protocol of this study was approved by the institutional review board of the three involved hospitals.

**Table 1:** Patient demographics in acute postsurgical and hematogenous PJI

	Acute postsurgical PJI (*n* = 34)	Acute hematogenous PJI (*n* = 67)	Total PJI (*n* = 101)	*p*-Value
Age (years)	75 ± 6.9	74 ± 9.2	74 ± 8.5	0.511
Female	26 (76)	55 (82)	81 (80)	0.503
BMI (kg/m^2^)	25.0 ± 3.4	27.7 ± 5.0	26.8 ± 4.6	**0.003**
Diabetes	9 (26)	39 (58)	48 (48)	**0.003**
CCI	3.6 ± 1.0	3.7 ± 1.4	3.6 ± 1.3	0.686
KLIC score	3.2 ± 1.3	3.6 ± 1.4	3.4 ± 1.4	0.198
Symptom duration (days)	6.8 ± 6.8	8.3 ± 8.2	7.8 ± 7.8	0.360
Operation				
Primary	30 (88)	59 (88)	89 (88)	1.000
Revision	4 (12)	8 (12)	12 (12)	
Implant age (Month)^*^	0.0 (0.0–1.0)	16.0 (8.0–43.5)	9.0 (1.0–35.0)	** < 0.001**
Preoperative CRP	11.0 ± 9.7	13.6 ± 10.5	12.72 ± 9.75	0.211
Type of organism				
*Staphylococcus aureus*	6 (18.2)	15 (22.4)	21 (21)	0.585
Antibiotic resistant (MRSA, MRSE)	8 (24.2)	9 (13.4)	17 (17)	
*Streptococcus*	5 (15.2)	15 (22.4)	20 (20)	
*Enterococcus*	1 (3.0)	1 (1.5)	2 (2)	
*Candida*	2 (6.1)	1 (1.5)	3 (3)	
Gram negative	2 (6.1)	9 (13.4)	11 (11)	
Polymicrobial	1 (3.0)	3 (4.5)	4 (4)	
Culture negative	8 (24.2)	14 (20.9)	22 (22)	

Data are presented as mean and standard deviation or number and percentage in parentheses

*PJI* periprosthetic joint infection, *n* number, *CCI* Charlson comorbidity index, *CRP* C-reactive protein, *MRSA* methicillin-resistant *Staphylococcus aureus*, *MRSE* methicillin-resistant *Staphylococcus epidermidis*

*Data of implant age are presented as median and interquartile range in parentheses

All surgeries were performed by seven surgeons in three centers. Even if DAIR techniques were not standardized between centers, all DAIR procedures included polyethylene insert change, complete resection of necrotic soft tissue, and massive irrigation using at least 9 L of saline. The surgical gloves were changed, and new draping was added before the surgical incision was closed. Postoperatively, intravenous antibiotics were administered for 4–6 weeks and then converted to oral antibiotic treatment.

Clinical evaluations were performed by fellowship-trained orthopedic surgeons in the three hospitals using the same methods. The patients were identified in the institutional databases using electronic medical records. Clinical information was collected by manual chart review. Patient demographics [age, sex, body mass index (BMI), Charlson Comorbidity Index (CCI), laterality, index surgery (primary or aseptic revision), Kidney, Liver, Index surgery, Cemented prosthesis and C-reactive protein value (KLIC) score, and DM] were investigated [20]. Symptom duration was defined as the time interval from first symptom onset to day of DAIR. Implant age was defined as the time interval between primary or revision surgery and day of DAIR. Preoperative level of serum C-reactive protein (CRP) was defined as the value checked at the first visit to the hospital for PJI-related symptoms. The causative organisms were classified into eight categories as follows: (1) *Staphylococcus aureus*, (2) antibiotic-resistant organisms including methicillin-resistant *Staphylococcus aureus* and methicillin-resistant *Staphylococcus epidermidis*, (3) *Streptococcus*, (4) *Enterococcus*, (5) *Candida*, (6) Gram-negative bacteria, (7) polymicrobial infections, and (8) negative cultures. DAIR failure was defined as a case where repeated DAIR or two-stage revision surgery was performed during the follow-up period. None of the patients included in the current study received chronic antibiotic suppression.

The overall success rate of DAIR and the factors related to DAIR failure were analyzed for both acute postsurgical and hematogenous infections. In addition, the factors related to DAIR failure in the hematogenous group were determined using subgroup analysis. Continuous variables including age, BMI, symptom duration, implant age, and preoperative serum CRP levels were described using mean and standard deviation (SD). Categorical variables including sex, CCI, DM, primary or revision TKA, acute postsurgical or hematogenous PJI, and type of organism were described using number and percentage. To compare demographics between the postsurgical group and the hematogenous group, a Student’s *t*-test was used for continuous variables and a chi-squared test was used for categorical variables. To determine the factors related to DAIR failure, a logistic regression test was used. Univariate analysis was performed with the independent variables of age, sex, BMI, DM, primary or revision surgery, symptom duration, implant age, preoperative serum CRP level, CCI, and acute postsurgical or hematogenous PJI. Then, multivariate analysis was performed using the variables with a *p*-value of < 0.2 on univariate analysis. The pattern of recurrence was analyzed using Kaplan–Meier survivorship curves with repeated DAIR or two-stage revision surgery as the endpoints.

## Results

The overall success rate of DAIR was 77%. On univariate analysis using the overall patient cohort, the success rate tended to be higher in the postsurgical group than in the hematogenous group (88% versus 72%; *p* = 0.060). Preoperative serum CRP levels also tended to be higher in the case of DAIR failure (*p* = 0.082) (Table 2). On multivariate analysis, these two factors did not reach statistical significance. The success rate tended to be higher in the postsurgical group than in the hematogenous group [odds ratio (OR) 2.443; confidence interval (CI) 0.839–8.496; *p* = 0.104]. In addition, the preoperative serum CRP levels tended to be higher in DAIR failure cases (OR 1.044; CI 0.998–1.094; *p* = 0.059).

**Table 2:** Results of univariate analysis for factors related to DAIR failure in overall patients

	Success (*n* = 78)	Failure (*n* = 23)	*p*-value
Age (years)	74.2 ± 8.6	73.4 ± 8.2	0.679
Female	63 (80.8)	18 (78.3)	0.772
BMI (kg/m^2^)	27 ± 4.9	26 ± 3.5	0.381
Diabetes	37 (47.4)	11 (47.8)	0.974
CCI	3.7 ± 1.4	3.5 ± 0.8	0.577
Symptom duration (days)	7.3 ± 7.6	9.3 ± 8.4	0.267
Operation			
Primary	71 (90)	19 (83)	0.462
Revision	8 (10)	4 (17)	
Implant age (Month)^*^	8.0 (1.0–33.0)	11.0 (5.0–35.5)	0.545
Preoperative CRP	11.6 ± 8.5	16.6 ± 12.5	0.082
Type of organism			
*Staphylococcus aureus*	15 (19.5)	6 (26.1)	0.139
Antibiotic resistant (MRSA, MRSE)	12 (15.6)	5 (21.7)	
*Streptococcus*	17 (22.1)	3 (13.0)	
*Enterococcus*	2 (2.6)	0 (0.0)	
*Candida*	2 (2.6)	1 (4.3)	
Gram negative	8 (10.4)	3 (13.0)	
Polymicrobial	1 (1.3)	3 (13.0)	
Culture negative	20 (26.0)	2 (8.7)	
Type of infection			
Acute postsurgical PJI	30 (38)	4 (17)	0.060
Acute hematogenous PJI	48 (62)	19 (83)	

Data are presented as mean and standard deviation or number and percentage in parentheses

*DAIR* debridement, antibiotics, and implant retention; *PJI* periprosthetic joint infection, *n* number, *CCI* Charlson comorbidity index, *CRP* C-reactive protein, *MRSA* methicillin-resistant *Staphylococcus aureus*, *MRSE* methicillin-resistant *Staphylococcus epidermidis*

^*^Data of implant age are presented as median and interquartile range in parentheses

There was no significant factor related to DAIR failure on subgroup analysis of acute hematogenous PJIs. On univariate analysis, only type of causative organism was related to increased DAIR failure (*p* = 0.035). Patients with antibiotic-resistant organisms and polymicrobial infections showed higher failure rates (Table 3). However, on multivariate analysis, no factor was associated with DAIR failure in the subgroup analysis of acute hematogenous PJIs.

**Table 3:** Results of univariate analysis for factors related to DAIR failure in patients with acute hematogenous PJI

	Success (*n* = 48)	Failure (*n* = 19)	*p*-Value
Age (years)	74.1 ± 9.4	72.6 ± 8.8	0.556
Female	39 (81.3)	16 (84.2)	1.000
BMI (kg/m^2^)	28.1 ± 5.4	26.6 ± 3.5	0.191
Diabetes	28 (58.3)	11 (57.9)	0.974
CCI	3.7 ± 1.6	3.5 ± 0.9	0.554
Symptom duration (days)	7.6 ± 7.8	9.8 ± 9.1	0.326
Operation			
Primary	44 (92)	15 (79)	0.209
Revision	4 (8)	4 (21)	
Implant age (Month)^*^	17.5 (8.8–47.8)	12.0 (7.0–38.0)	0.313
Preoperative CRP	12.3 ± 8.8	16.9 ± 13.6	0.186
Type of organism			
*Staphylococcus aureus*	10 (20.8)	5 (26.3)	0.035
Antibiotic-resistant (MRSA, MRSE)	5 (10.4)	4 (21.1)	
*Streptococcus*	13 (27.1)	2 (10.5)	
*Enterococcus*	1 (2.1)	0 (0.0)	
*Candida*	0 (0.0)	1 (5.3)	
Gram negative	7 (14.6)	2 (10.5)	
Polymicrobial	0 (0.0)	3 (15.8)	
Culture negative	12 (25.0)	2 (10.5)	

Data are presented as mean and standard deviation or number and percentage in parentheses

*DAIR* debridement, antibiotics, and implant retention, *PJI* periprosthetic joint infection, *n* number, *CCI* Charlson comorbidity index, *CRP* C-reactive protein, *MRSA* methicillin-resistant *Staphylococcus aureus*, *MRSE* methicillin-resistant *Staphylococcus epidermidis*

^*^Data of implant age are presented as median and interquartile range in parentheses

**Fig. 1:**
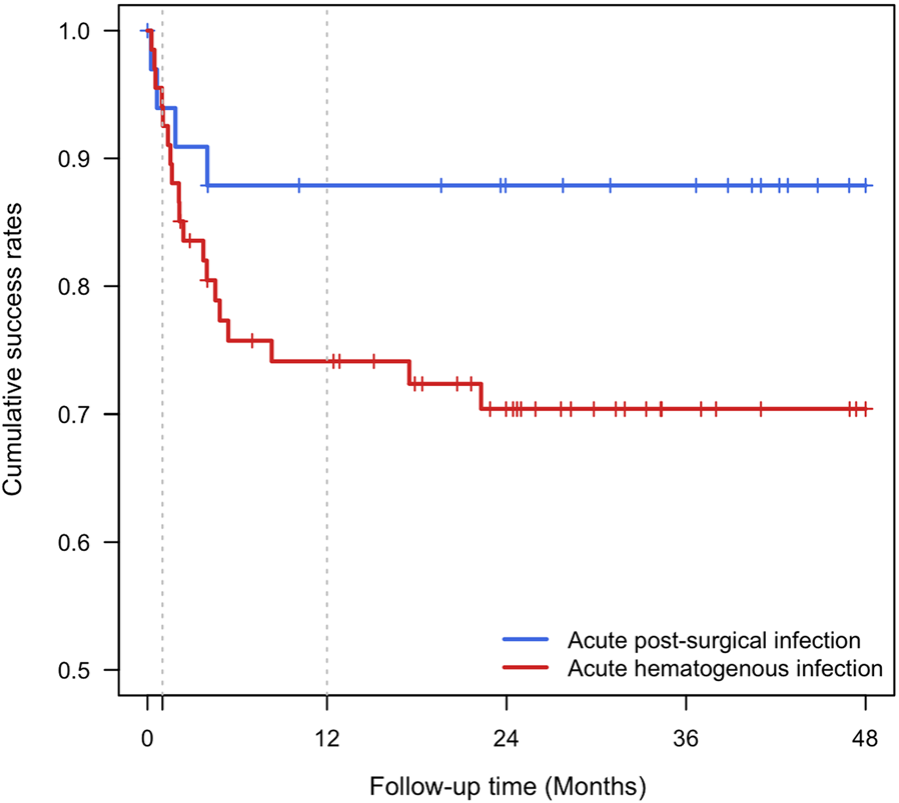
Pattern of recurrence analyzed using Kaplan–Meier survivorship curves in the postsurgical group and the hematogenous group

PJI recurrence occurred for a longer period of time after DAIR in the hematogenous group than in the postsurgical group. In the postsurgical group, the recurrence of PJI occurred until 3 months, whereas in the hematogenous group, recurrence occurred for up to 2 years (Fig. 1).

## Discussion

Despite the improvements in surgical techniques and the increased understanding of pathophysiology in PJI, the success rate of DAIR has not improved significantly. For successful DAIR, patient selection is probably the most important factor, and especially in acute hematogenous PJIs, there may be more considerations than for acute postsurgical PJIs. In the present study, the failure rate of DAIR tended to be higher in patients with acute hematogenous PJIs than with acute postsurgical PJIs. Furthermore, on subgroup analysis using only acute hematogenous PJIs, the causative organism was related to increased DAIR failure in the univariate analysis. However, no factor related to DAIR failure in acute hematogenous PJIs was identified on multivariate analysis. In terms of PJI recurrence, there was a case of late recurrence up to 2 years after DAIR in the hematogenous group, which means that closer observation is required for a longer period of time in patients with acute hematogenous PJIs even if it is judged that the hematogenous PJI is successfully controlled.

The differences in DAIR success rates for PJI of the knee between previous studies can be attributed to the heterogeneity of the studies. Previous studies included both hip and knee PJIs or included patients with or without polyethylene insert changes [8, 11]. PJI of the knee is a serious but not common complication, and it occurs in 1–2% of the cases, making prospective studies difficult [21]. Also, the DAIR success rate may depend upon the surgical technique and perioperative management, and these factors may be the reason the surgical results differed between single-center studies [5, 22]. Therefore, research related to the success rate of DAIR should be conducted in a multicenter study with relatively larger numbers of subjects. Although a recent study reported a relatively low DAIR success rate of 64% in patients with early postoperative PJI, other studies reported that it was close to 80% [5, 8]. In our study, the overall success rate was 77%. To increase the success rate of DAIR, studies have reported the factors related to failure, and further studies are needed to determine if substantial improvements in success rates can be obtained in the future.

One of the findings that can be relatively useful in determining whether to perform DAIR surgery is that acute postsurgical PJIs have been reported to have a higher success rate than hematogenous PJIs [8, 17]. It was reported that DAIR was successful in 64% of early PJIs (implant age < 1 year) versus 38% of late hematogenous PJIs (implant age > 1 year; OR 1.78; *p* = 0.01) [8]. One possible explanation is that the elapsed time from infection to DAIR is one of the important factors in DAIR success, and the duration of symptoms is clearer in acute postsurgical PJIs than in acute hematogenous PJIs. In addition, in hematogenous PJIs, not only is the duration of symptoms ambiguous, but there could also be cases of acute exacerbation by chronic infection. A possible reason for the less clear symptom duration in acute hematogenous PJIs is that the virulence of the causative organism is low, and the symptoms are not severe, so early infection detection may be difficult. However, there was no difference in the causative organisms in the patients in our study. If such a difference is present, there may be cases where severe symptoms do not appear or the patients are not able to recognize them. This may be related to reduced inflammatory responses or pain sensations. These may occur in patients who are chronic users of nonsteroidal anti-inflammatory drugs or pain killers, or in patients with neuropathy. The prevalence of DM of the acute hematogenous PJI group in our study was higher than in the acute postsurgical infection group (58% versus 26%). Whether this was directly related to increased DAIR failure, or whether it delays the time of infection detection owing to other causes such as polyneuropathy, ultimately worsening the outcome of DAIR, needs to be studied further.

It is important to know which factors in patients with hematogenous PJIs are associated with recurrence. It was revealed that *Staphylococcus aureus* and Gram-negative infections were risk factors for DAIR failure in PJIs with an implant age greater than 1 year [8]. Univariate analysis on subgroup analysis using only hematogenous infections in our study showed similar results, that the causative organism was related to increased DAIR failure in hematogenous PJIs. However, in our study, antibiotic-resistant organisms and polymicrobial infections were associated with DAIR failure. Our findings are supported by a recent study reporting that the presence of DM and polymicrobial infections was related to DAIR failure in acute hematogenous PJIs of hip and knee joints [23]. It is noteworthy that recurrences were not more common in Gram-negative bacteria and fungal PJIs in the present study. Since the number of PJIs caused by these atypical organisms was relatively small, further studies are necessary to confirm these findings.

Our findings support the hypothesis that recurrence patterns differ between acute postsurgical and acute hematogenous PJIs. Xu et al. reported an increased failure of DAIR over time after surgery [11]. In their study, the 1-year failure rate was 41.2% whereas the failure rate at 2 years was 45.9%. Another study comparing the failure rate of DAIR between acute postsurgical and hematogenous PJIs showed that acute hematogenous PJIs had consistent recurrences during a 1-year follow-up period, whereas most acute postsurgical PJIs recurred at an early stage [17]. The present study also showed recurrences of infections for up to 2 years in the hematogenous group, consistent with previous studies. Therefore, continuous and close follow-up including long-term antibiotic suppression is required even if it is judged that the hematogenous PJI is successfully controlled using DAIR [24].

There were several limitations to this study that should be considered. First, the sample size was relatively small. Since the occurrence of PJI after arthroplasty surgery is rare, even though the study was conducted in a multicenter study, for some variables, only tendencies could be confirmed rather than clear statistical significance identified on subgroup analysis. This needs to be confirmed in further studies with higher numbers of subjects. Second, to include as many patient samples as possible, patients were enrolled over a long period of time, so the PJI diagnostic criteria used in this study were different according to the time period. However, the MSIS criteria were used after 2011. In addition, patients diagnosed with PJI before 2011 were retrospectively confirmed as PJI by applying the same criteria. Third, depending upon each surgeon or hospital, the surgical method may have differed, such as whether betadine scrubbing or soaking was used. In addition, duration of antibiotic treatments was different between patients according to the patients’ conditions and causative organisms. However, this may be an advantage of a multicenter study that reflects real-world practice. In addition, all surgeries were performed following the principles of polyethylene change, thorough debridement of necrotic tissue, and massive irrigation, which are at the core of the DAIR procedure.

## Conclusion

The failure rate tended to be higher in the acute hematogenous PJI group compared with the acute postsurgical PJI group, even if no definite factor related to DAIR failure for acute hematogenous PJIs was identified. Since acute hematogenous infections may recur for a longer period after DAIR than postsurgical infections, caution and careful follow-up are required.

## Data Availability

Not applicable.

## Abbreviations

PJI: Periprosthetic joint infection
DAIR: Debridement, antibiotics, and implant retention
TKA: Total knee arthroplasty
MSIS: Musculoskeletal Infection Society
DM: Diabetes mellitus
BMI: Body mass index
CCI: Charlson comorbidity index
CRP: C-reactive protein
